# Inpatient Care Costs of COVID-19 in South Africa’s Public Healthcare System

**DOI:** 10.34172/ijhpm.2021.24

**Published:** 2021-04-25

**Authors:** Ijeoma Edoka, Heather Fraser, Lise Jamieson, Gesine Meyer-Rath, Winfrida Mdewa

**Affiliations:** ^1^SAMRC Centre for Health Economics and Decision Science-PRICELESS SA, School of Public Health, Faculty of Health Sciences, University of the Witwatersrand, Johannesburg, South Africa.; ^2^Health Economics and Epidemiology Research Office, Department of Internal Medicine, School of Clinical Medicine, Faculty of Health Sciences, University of the Witwatersrand, Johannesburg, South Africa.; ^3^Department of Global Health, School of Public Health, Boston University, Boston, MA, USA.

**Keywords:** Inpatient Cost, COVID-19, South Africa, Healthcare Budget, Economic Cost, Financial Cost

## Abstract

**Background:** Coronavirus disease 2019 (COVID-19) has had a devastating impact globally, with severe health and economic consequences. To prepare health systems to deal with the pandemic, epidemiological and cost projection models are required to inform budgets and efficient allocation of resources. This study estimates daily inpatient care costs of COVID-19 in South Africa, an important input into cost projection and economic evaluation models.

**Methods:** We adopted a micro-costing approach, which involved the identification, measurement and valuation of resources used in the clinical management of COVID-19. We considered only direct medical costs for an episode of hospitalisation from the South African public health system perspective. Resource quantities and unit costs were obtained from various sources. Inpatient costs per patient day was estimated for consumables, capital equipment and human resources for three levels of inpatient care – general wards, high care wards and intensive care units (ICUs).

**Results:** Average daily costs per patient increased with the level of care. The highest average daily cost was estimated for ICU admissions – 271 USD to 306 USD (financial costs) and ~800 USD to 830 USD (economic costs, excluding facility fee) depending on the need for invasive vs. non-invasive ventilation (NIV). Conversely, the lowest cost was estimated for general ward-based care – 62 USD to 79 USD (financial costs) and 119 USD to 278 USD (economic costs, excluding facility fees) depending on the need for supplemental oxygen. In high care wards, total cost was estimated at 156 USD, financial costs and 277 USD, economic costs (excluding facility fees). Probabilistic sensitivity analyses suggest our costs estimates are robust to uncertainty in cost inputs.

**Conclusion:** Our estimates of inpatient costs are useful for informing budgeting and planning processes and cost-effectiveness analysis in the South African context. However, these estimates can be adapted to inform policy decisions in other context.

## Background

**Key Messages**

**Implications for policy makers**

- Inpatient care cost of coronavirus disease 2019 (COVID-19) vary by ward type in South Africa’s public hospitals.
- Average daily costs per patient is highest for intensive care, and the lowest for general ward-based care.
- These estimates are useful for budgeting and planning as well as important inputs for the cost-effectiveness analysis of COVID-19 interventions.

**Implications for the public**

This study estimates inpatient care costs of managing severe and critical cases of coronavirus disease 2019 (COVID-19) in South Africa’s public healthcare system. Financial cost estimates from this study are a useful input for estimating national budgets for planning purposes. Furthermore, our estimates of economic costs are important inputs for cost-effectiveness analysis assessing the value for money of therapeutic and preventative strategies against COVID-19 to ensure efficient allocation of scarce resources.

Coronavirus disease 2019 (COVID-19), an infectious disease caused by the novel severe acute respiratory syndrome coronavirus 2 (SARS-CoV-2), was first discovered in December 2019 following an outbreak in the Hubei province of China. By the beginning of March 2020, it had spread to several countries globally and on 11th March was declared a pandemic by the World Health Organization (WHO).^1^ COVID-19 has since had a devastating impact globally, with severe health and economic consequences on individuals and households.^2^

 In South Africa, the first case was reported on March 5, 2020. To forestall the spread of the disease, while preparing the health system to deal with potential surges in severe and critical cases,^3^ a nationwide lockdown was initiated on March 24, 2020 for approximately three months. During this period, a national COVID-19 costing working group was convened to support the National Department of Health (NDOH) in estimating a budget to prepare the public health system’s response to the pandemic. A national COVID-19 budget model was developed^4^ to predict extra resources required by the public health system during the pandemic using epidemiological projections of the number of cases and hospitalisations under different scenarios from the National COVID-19 Epi Model.^5^ This study estimates inpatient care for managing COVID-19 patients in South Africa’s public hospitals, an important input into the national COVID-19 budget model.

 The South African NDOH guidelines for the clinical management of COVID-19 disease recommend different care pathways for patients with varying degrees of respiratory distress and complications from COVID-19.^6^ This includes management of hypoxemic respiratory failure using supplemental oxygen therapy delivered via nasal cannula or face masks for severe cases, and the management of severe hypoxemic respiratory failure using either non-invasive ventilation (NIV) and/or mechanical ventilation for critical cases. Given different quantities of resource requirements at each level of clinical care, costs per day is expected to differ across care levels. Thus, we estimated cost per patient per day for each clinical care level from the public health system perspective, accounting for a wide range of resources, broadly grouped into three categories – consumables, capital equipment and human resources. We distinguished between financial costs, which reflect the direct expenditure on goods and services needed within the healthcare system, and economic costs, which reflect opportunity costs and captures the full value of all resources deployed for the inpatient care of COVID-19 in the public health sector.^7^ The former (financial costs) are useful for budgeting and planning purposes while the latter (economic costs) are important inputs into economic evaluations assessing the cost-effectiveness of treatments/clinical care regimens for COVID-19 as well as the cost-effectiveness of COVID-19 vaccines.^8^

## Methods

###  Study Design 

 A micro-costing approach was used to estimate average daily inpatient cost of managing severe and critically ill COVID-19 cases in South Africa. This involves three steps – first, the identification of all resources used for an episode of hospitalisation with COVID-19; second, the measurement of the quantities of each resource input; and finally the valuation of each input using appropriate unit prices. We adopted the Global Health Costing Consortium Reference Case for Estimating Cost of Global Health Services and Interventions to reflect best practices in costing.^7^ We considered only direct medical costs incurred by individuals of all ages during an episode of hospitalisation, from the South African public healthcare system perspective. Inpatient costs per patient day were estimated for three broad cost categories (consumables, capital equipment and human resources costs – Table 1) and for three different levels of care – care provided within general wards, high care wards and care provided within intensive care units (ICUs). We assume that care requiring supplemental oxygen therapy are managed in general wards while inpatient care requiring higher levels of respiratory support are either managed with high flow nasal oxygen (HFNO) in high care wards or with continuous positive airway pressure (CPAP), bi-phasic NIV or invasive mechanical ventilation (IMV) in ICUs.

**Table 1:** Cost Categories and Resource Inputs

**Cost Category**	**Resource Inputs**
**Consumables: **Items or equipment that are non-durable, cannot be reused and/or need regular replacement with a useful life of less than a year	
1. Therapeutic agents	Antibiotics/antimicrobials, corticosteroids (dexamethasone), nutritional support (parenteral nutrition) and anticoagulation
2. Management of complications	Sodium chloride 0.9% and vasoconstrictive agents (for septic shock), Renal replacement therapy (for acute kidney injury), lactulose (for acute liver failure), primary percutaneous coronary intervention, fibrinolysis, analgesics & opioids (for acute cardiac injury) and intercostal drain (for pneumothorax)
3. Diagnostic tests	Chest X-ray, full blood count, urea, C-reactive protein, erythrocyte sedimentation rate, blood gas analysis, HIV, tuberculosis sputum microscopy, culture and susceptibility & SARS-CoV-2 polymerase chain reaction test
4. Respiratory support and accessories	Oxygen flow, oxygen face mask, nasal cannula, high flow machine consumables, CPAP mask (cushioned anaesthetic mask), NIV mask (cushioned anaesthetic mask), suction catheters; oropharyngeal airway; endotracheal tube
5. PPE	Non-sterile gloves, goggles, visors, plastic aprons, cotton gowns with apron, surgical face masks, N95 respirator, water resistant gowns
6. Hygiene infection and control	Hand rub, liquid hand soap, paper towels, 70% alcohol disinfectant (for disinfection of equipment), 0.5% sodium hypochlorite (for surface disinfection)
**Capital equipment: **Any resource that has a useful life of more than one year	
1. Medical devices	Patient monitor, infusion pump
2. Respiratory support accessories	Oxygen Concentrator, high flow machine, suction pump, backup oxygen cylinder, Ambu-bag (self-inflating bag), Laryngoscope
3. Ventilator	CPAP machine, biphasic NIV machine, mechanical ventilator
4. Beds	Manual hospital beds and mattresses
5. Bed Linen	Blanket, draw sheet, bed sheet, mattress protector, blanket cover patient suits, pillow cover, dressing gowns, pillows
**Human resources: **Clinical and non-clinical staff responsible for the care of COVID-19 patients	
1. Medical staff	Physicians/internal medicine physician registrars and intern, intensivists, consultant (anaesthetist/internal medicine), cardiovascular specialists, pulmonologists
2. Allied health professionals	Infection control officers, respiratory physiotherapists, radiographer, social worker, medical technologists, nutritionist, clinical pharmacist
3. Nurses	Critical care nurses, professional nurses, staff nurses
4. Support staff	Administrative staff, cleaning staff

Abbreviations: CPAP, continuous positive airway pressure; NIV, non-invasive ventilation; COVID-19, coronavirus disease 2019; SARS-CoV-2, severe acute respiratory syndrome coronavirus 2; PPE, personal protective equipment.

 To identify the types of resource inputs used for the clinical care management of COVID-19 patients, we used South Africa-specific guidelines, including the NDOH and Critical Care Society of Southern Africa guidelines.^6,9^ These local guidelines were also used as a source of information on resource quantities. Additional resource quantities were obtained through a literature search conducted on Google Scholar and PubMed on March 31, 2020 and updated on August 18, 2020. Search terms included “clinical management of COVID-19;” “management of hospitalised patients with COVID-19;” “clinical outcomes of COVID;” “management of complications in hospitalised patients with COVID;” “personal protective equipment requirements for COVID;” “infection prevention and control for hospitalised COVID-19 patients;” “costs associated with management of severe pneumonia,” and “personnel requirements for clinical management of COVID.” Studies selected include those that reported on the clinical management of confirmed COVID-19 cases, severe acute respiratory syndrome (SARS) and Middle East respiratory syndrome coronavirus (MERS-CoV) within a hospital setting. We reviewed studies published in English from China, the United States, the United Kingdom, Italy, and South Africa. Studies were selected based on topic relevance and quality of evidence (judged based on study design). Where available, systematic reviews were used, failing which individual randomised clinical trials and observational studies were included. Each resource input was valued using unit prices obtained from South African public-sector sources. These include the National Health Laboratory Service price list, the Master Procurement Catalogue, the Uniform Patient Fee Schedule, the National COVID-19 Budget Model and tender documents.

 Finally, average cost per patient per day in each hospital ward was estimated as the sum of daily per patient consumable, capital equipment and human resource costs. For economic costs, average cost per patient was estimated as the sum of all three cost categories while financial cost included only daily costs of consumables and capital equipment based on the assumption of no incremental expenditure for additional staff. Costs were converted from South African Rand (ZAR) and expressed in 2020 US Dollars (USD), using an exchange rate of USD to 16.61 ZAR.^10^

 A detailed description of the estimation of each resource input broadly grouped under the three broad categories is provided in Table 1.

###  Consumables

 We define consumables as items or equipment that are non-durable, cannot be reused and/or need regular replacement. We reviewed South African COVID-19 clinical management guidelines, published articles and made use of expert opinions to identify the various types of consumables required and their quantities. Resource inputs identified as consumables include diagnostic tests, therapeutic agents, respiratory support (and accessories), personal protective equipment (PPE), and cleaning agents for hygiene infection and control (Table 1). To estimate the average quantity of each resource input per day, we multiplied the quantity of resource inputs required per day by the proportion of patients requiring each input. Where a resource item was required only once during the duration of hospitalisation, the quantity was divided by the average length of stay within the respective wards to estimate the resource used per day. We used average (min–max) length of stay obtained from the South African COVID-19 Hospital Sentinel Surveillance database^11^: 8 (4–12) days for the general ward admissions; 7 (3–13) days for high care and ICU admissions with CPAP or NIV; and 16 (10–31) days for ICU admissions with IMV.

 Finally, unit prices for each item were applied to average quantity required per patient per day to estimate a cost per day for each item. Unit prices for consumables were obtained from South African government commodity tenders,^12,13^ government procurement documents,^14-16^ the National COVID-19 Budget Model South Africa,^4^ published studies^17,18^ and retail advertisements.^16-18^ A detailed description of average daily quantities, unit prices and average per patient daily costs of each consumable is provided in Table S1-S6 of Supplementary files 1 and 2.

###  Capital Equipment

 In this study, we defined capital equipment as any resource that has a useful life of more than one year. Resources that fall into this category include hospital equipment, beds and bed linen (Table 1). Details of each resource, their quantities and unit prices are provided in Table S7 of Supplementary file 3. The quantity of each capital item of equipment required per day and proportion of patients needing an item was based on the WHO COVID-19 Essential Supplies Forecasting Tool^22^ and expert opinion. We assumed that hospital equipment and beds had a useful life of 10 years and hospital linen, 5 years. To estimate the economic cost of capital equipment, we first estimated equivalent annual costs for all capital equipment by dividing the unit price of the equipment by an annuitisation factor, calculated as 7.72% and 4.34% for equipment with a 10 year and 5-year life span respectively, based on a discount rate of 5%.^23,24^ To estimate annual financial costs of capital equipment, we used the straight-line depreciation approach by dividing the unit price of the capital equipment by its estimated useful life. The unit prices of capital equipment were obtained from the South African National COVID-19 Budget Model^4^ and retail advertisements.^25-29^

 Finally, daily equipment costs (financial and economic) were estimated. For daily financial costs, annualised financial cost was divided by 365 days. However, for daily economic costs, we assumed the equipment would be used for two thirds of the year (nine months) for patients with COVID-19. This assumption was tested in a sensitivity analysis where period of use was varied between 4 and 12 months.

###  Human Resources

 Human resource requirements for the different levels of care (Table 1 and Table S8 of Supplementary file 3) were obtained from the Critical Care Society of Southern Africa guidelines and from expert opinion.^30^ Staff-to-patient ratios were based on the opinions of government experts working at appropriate staffing levels of care for COVID-19. Exclusive of overtime hours, we assumed that staff worked an average of 1720 hours annually, which was used to estimate the hourly wage per cadre. The number of hours worked per day by each staff in a ward was multiplied by the probability that the staff would provide care to patients, and by the staff-to-patient ratio, to estimate the staff time per patient per day. Finally, staff time per patient per day was multiplied by daily staff remuneration rate to estimate the staff daily cost per patient. Staff remuneration and benefits were obtained from the South African Department of Public Service and Administration salary scales^31^ which is made up of monthly salary, overtime and any benefits that are applicable.

###  Overheads

 In addition to the cost categories laid out above, ward overhead costs were also considered in this study. This was included as the median facility fees of each hospital ward (Table S9 of Supplementary file 3) obtained from the Uniform Patient Fee Schedule.^32^ Facility fees are described by the NDOH as the “cost of providing an environment within which a health service can be delivered”^32^ and may have included some resources already accounted for under our three cost categories. Therefore, we present our final costs estimates inclusive and exclusive of facility fees.

###  Sensitivity Analysis

 Given uncertainties in some of our resource input quantities and assumptions, we conducted a series of both scenario and probabilistic sensitivity analyses to assess the robustness of our final cost estimates to uncertainty in our inputs. For the scenario analyses, we assessed uncertainties in the estimates of PPE quantities required, hospital length of stay, staff-to-patient ratios, and number of bed-days per year for COVID-19 patients. The ranges over which each input was varied are provided in Supplementary files 1-3.

 A probabilistic sensitivity analysis (PSA) was conducted to simultaneously assess the robustness of our final cost estimates to uncertainty in multiple inputs. The PSA was performed by fitting appropriate probability distributions to each input and running 1000 Monte Carlo simulations that draws randomly from these distributions.^33,34^ The parameters that were varied in this way include length of stay in each hospital ward; the proportion of patients requiring different therapeutic agents and diagnostic test; and the incidence of complications.

## Results

Tables 2-4 displays estimates of daily cost per patient for consumables, capital equipment and human resources in each hospital ward – general wards (with and without supplemental oxygen therapy), high care wards and ICUs.

**Table 2:** Consumables Daily Costs per COVID-19 Patient (2020 USD)

**Resources**	**General Ward**	**High Care Ward**	**ICU-CPAP**	**ICU-NIV**	**ICU-IMV**
Therapeutic agents	3.87^a^/4.44^b^	5.91	53.70	53.70	53.70
Management of complications	-	-	92.91	92.91	54.11
Diagnostics	21.14	20.14	16.70	32.86	32.56
Respiratory support	16.19^b^	73.67	48.86	48.83	49.15
PPE	33.35	50.03		66.71	
Hygiene and Infection control	2.72	4.03		5.33	
Total consumables	61.10^a^/77.85^b^	153.79	284.21	300.34	261.55

Abbreviations: COVID-19, coronavirus disease 2019; USD, United States Dollar (1 USD = 16.61 ZAR); CPAP, Continuous positive airway pressure ventilation; NIV, Non-invasive ventilation; IMV, Invasive mechanical ventilation; ICU, Intensive care unit; PPE, personal protective equipment. ^a^ Cost in general ward without oxygen support. ^b^ Cost in general ward with oxygen support.

**Table 3:** Capital Equipment Daily Costs Per COVID-19 Patient (2020 USD)

**Equipment Costs**	**Economic Costs**					**Financial Costs **				
	**General Ward**	**High Care Ward**	**ICU-CPAP**	**ICU-NIV**	**ICU-IMV**	**General Ward**	**High Care Ward**	**ICU-CPAP**	**ICU-NIV**	**ICU-IMV**
Medical devices	0.51	0.57	1.54	1.54	1.54	0.26	0.30	0.80	0.80	0.80
Respiratory support accessories	1.22^a^	3.22	1.35	1.35	1.55	0.63^a^	1.66	0.69	0.69	0.81
Ventilator	-	-	5.14	6.89	14.37	-	-	2.65	3.55	7.39
Bed and bed linen	0.70	0.70	0.70	0.70	0.70	0.38	0.38	0.38	0.38	0.38
Total equipment cost	1.21^b^/2.43^a^	4.49	8.73	10.48	18.16	0.64^b^/1.28^a^	2.34	4.52	5.42	9.38

Abbreviations: COVID-19, coronavirus disease 2019; USD, United States Dollar (1 USD = 16.61 ZAR); CPAP, Continuous positive airway pressure ventilation; NIV, Non-invasive ventilation; IMV, Invasive mechanical ventilation; ICU, Intensive care unit. ^a^ Cost in general ward with oxygen support. ^b^ Cost in general ward without oxygen support.

**Table 4:** Human Resource Daily Costs per COVID-19 Patient (2020 USD)

**Staff Cadre**	**General Ward**	**High Care Ward**	**Intensive Care Unit**
Medical staff (doctors)	26.16	21.57	37.79
Allied health professionals	1.15	13.00	31.77
Nurses	28.12	81.07	444.08
Support staff	1.74	3.88	4.53
Total	57.17	119.52	518.17

Abbreviation: COVID-19, coronavirus disease 2019.

###  Consumables Costs

 Daily consumables cost per patient increased with higher levels of care (Table 2). The exception to this was in inpatient care requiring IMV. Due to the longer length of stay in ICU with IMV, consumables that were required only once during a hospital episode (mainly primary percutaneous coronary intervention for the management of acute cardiac injury) had a lower daily cost, consequently, resulting in a lower overall cost of consumables compared to inpatient care requiring CPAP or NIV in ICUs. Variations in average quantity required per day (as detailed in Tables S1-S6) drove the difference in cost per patient per day in the different care scenarios. In general wards, the largest cost driver was daily PPE costs (Table 2), accounting for approximately half of total consumable costs in general wards. In high care wards, the largest cost driver was respiratory support, reflecting the higher daily volume of oxygen required for HFNO therapy (Table 2). Finally in ICU, the highest contributor to consumables cost was observed to be the cost of managing complications^[1]^ and PPE cost, both collectively contributing to approximately half the total consumables cost in ICU.

###  Capital Equipment Costs

Table 3 presents capital equipment daily costs per patient – both economic costs and financial costs. Similar to consumables, daily per patient costs of capital equipment (both financial and economic costs) increased with the level of hospital care provided (Table 3). Daily cost per patient was highest in ICUs for care requiring invasive mechanical ventilators (~18 USD, economic costs; ~9 USD, financial costs). This was largely due to daily costs of mechanical ventilators.

###  Human Resource Costs

 Average human resource costs were estimated at ~518 USD for inpatient care in ICUs, ~120 USD for care in high care wards and 57 USD for care in general wards (Table 4). The higher costs observed in ICU was largely driven by the higher number of staff cadre types required for the management of critical patients and the higher staff-to-patient ratios in ICUs (Table S8). Across all ward types, nursing staff costs was observed to be the highest cost driver (Table 4).

###  Total Costs

 Total average cost per patient per day (both economic and financial costs) for each hospital ward is presented in Table 5. Overall, the highest average costs (economic) were observed for inpatient care in ICUs, ranging from ~800 USD to ~830 USD, exclusive of facility fees and from ~1314 USD to ~1345 USD when facility fees are included (Table 5). The lowest cost was observed in general ward-based care without supplemental oxygen therapy – 119 USD increasing to 184 USD when facility fees are included. Overall, consumable costs accounted for the highest proportion of total daily costs in general and high care wards while human resource costs accounted for the highest proportion of total daily costs in ICUs (Table 5).

**Table 5:** Total Daily Costs Per COVID-19 Patient (2020 USD)

**Ward Type**	**Respiratory Support**	**Economic Costs **					**Financial Costs**		
		**Total Average Costs**		**Totals by Cost Category** ** [% Of Total Costs]**			**Total Costs**	**Totals by Cost Category** ** [% Of Total Costs]**	
		**Exclusive Facility Fee**	**Inclusive Facility Fee**	**Consumables**	**Human Resources**	**Equipment **	**Exclusive** ** Facility Fee**	**Consumables**	**Equipment**
General ward	None	119.47	184.05	61.10 [51%]	57.17 [48%]	1.21 [1%]	61.74	61.10 [99%]	0.64 [1%]
	Supplemental oxygen	137.45	202.03	77.85 [57%]	57.17 [42%]	2.43 [2%]	79.13	77.85 [98%]	1.28 [2%]
High care ward	HFNO	277.80	474.35	153.79 [55%]	119.52 [43%]	4.49 [2%]	156.12	153.79 [99%]	2.34 [1%]
ICU	CPAP	811.11	1327.46	284.21 [35%]	518.17 [64%]	8.73 [1%]	288.72	282.21 [98%]	4.52 [2%]
	NIV	828.99	1345.35	300.34 [36%]	518.17 [63%]	10.48 [1%]	305.76	300.34 [98%]	5.42 [2%]
	IMV	797.89	1314.24	261.55 [33%]	518.17 [65%]	18.16 [2%]	270.94	261.55 [97%]	9.38 [3%]

Abbreviations: COVID-19, coronavirus disease 2019; USD, United States Dollars (1 USD=16.61 ZAR); CPAP, continuous positive airway pressure ventilation; NIV, non-invasive ventilation; IMV, invasive mechanical ventilation; ICU, Intensive care unit; HFNO, high flow nasal oxygen.

###  Sensitivity Analysis 

 The results of the scenario analysis suggest that the uncertainty in the staff-to-patient ratio had the largest impact on economic cost per patient per day (Figures S1-S6 in Supplementary file 4). For example, in general wards without supplemental oxygen, when the staff-to-patient ratio was assumed to be 50% higher than the base estimates, economic cost increased from 119 USD to 175 USD per patient per day. Conversely, economic cost per day decreased from 119 USD to 100 USD when staff-to-patient ratio was assumed to be 50% lower (Figure S1B). Uncertainty in other inputs including quantities of PPE and hygiene and infection control resources, length of hospital stay, and number of bed days per year also impacted on our economic cost estimates, but to a lesser extent (Figures S1B-S6B in Supplementary file 4).

 For financial costs, uncertainty in PPE quantities used had the largest impact on general ward (Figures S1A and S2A), high care ward (Figure S3A), and ICU with IMV costs (Figure S6A). For ICU costs with CPAP or NIV, uncertainty in length of stay had the largest impact on average cost per patient per day (Figures S4A and S5A).

 The results of the PSA are presented as box plots (Figure). The results show that, even with the maximum and minimum cost estimates from the PSA, uncertainty in cost inputs resulted in a variability of less than 20 USD for general ward and high care ward daily costs (Figure). For ICU costs, although uncertainty in our cost inputs resulted in wider variability over the whole distribution of costs estimates produced from the PSA, the interquartile range was still well within 20 USD across all ICU scenarios (Figure). Overall, our cost estimates are robust to joint uncertainty in the length of hospital ward stay, the proportion of patients requiring different therapeutic agents and diagnostic tests as well as uncertainty in the incidence of complications.

**Figure:**
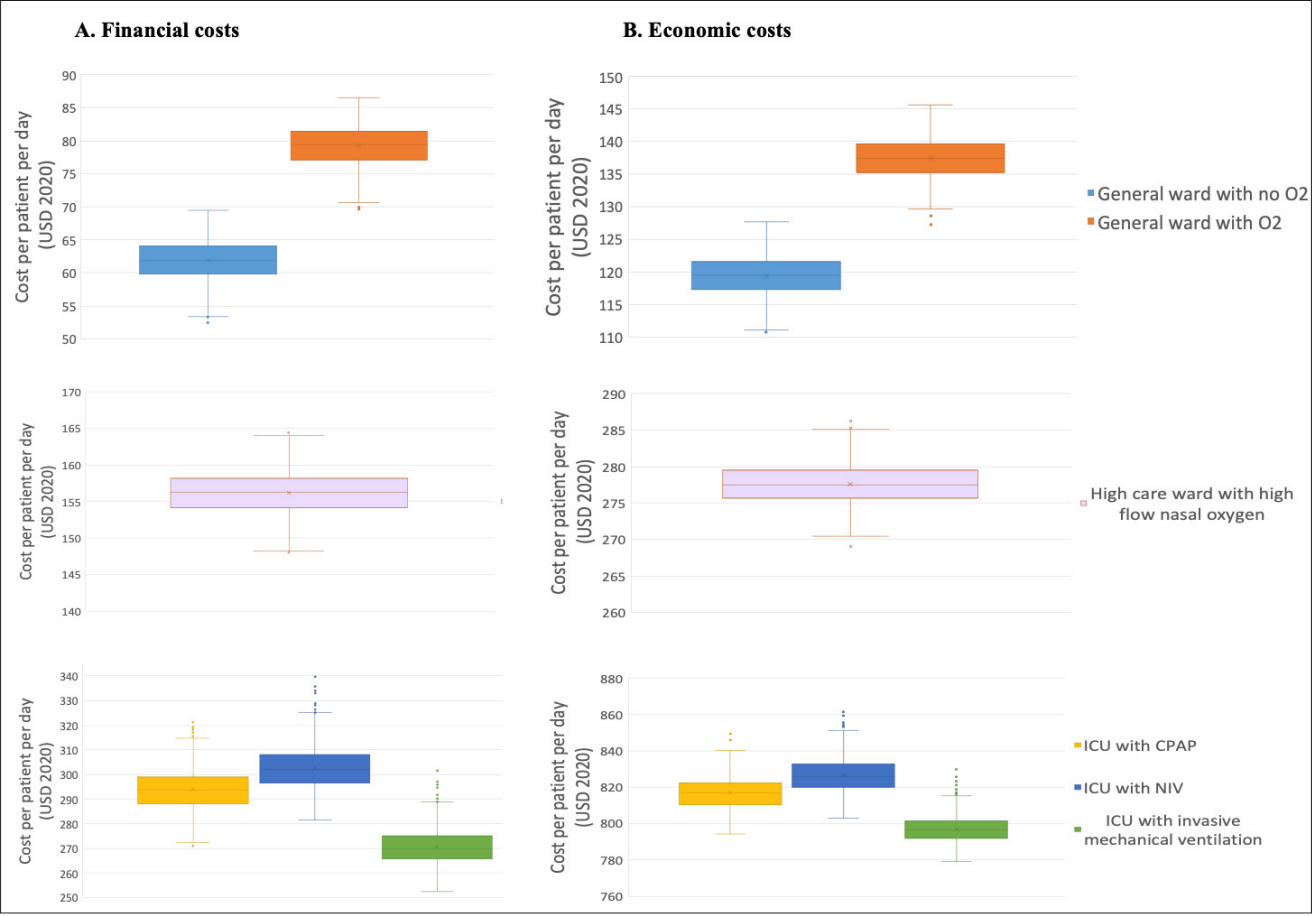
PSA Showing the Range of Average Daily Cost Per Patient (Excluding Facility Fees). Abbreviations: PSA, Probabilistic Sensitivity Analysis; ICU, Intensive care unit; CPAP, continuous positive airway pressure; NIV, non-invasive ventilation.

## Discussion

 In the wake of the global COVID-19 pandemic, several strategies were implemented to manage severe and critical cases of COVID-19. This study estimates average daily costs of inpatient care of COVID-19 in South Africa’s public hospitals. The COVID-19 pandemic presents significant challenges to healthcare systems around the world, not least to health systems in low- and middle-income countries facing higher resource constraints. Therefore, difficult decisions have to be made on how best to allocate scarce public resources to maximise population health during the pandemic. For example, should resources be spent scaling up ICU capacity and the provision of high level non-invasive and/or invasive oxygen therapies, or should resources be allocated to the expansion of lower level health infrastructure such as general ward capacity? Cost-effectiveness analysis that compares the costs and benefits of competing use of scarce resources can be a useful tool for answering these questions. Cost estimates presented in this study are important inputs for cost-effectiveness analysis of COVID-19 interventions. However, in addition to these cost estimates, such cost-effectiveness analysis will require estimates of the effectiveness of each clinical care regimens, typically obtained using robust data collection methods such as randomised control trials. Although other data sources such as observational data may provide useful insights into outcomes of different clinical care regimens, observational data may be subject to selection bias due to non-random sampling which could bias estimates of the clinical effectiveness of an intervention.^37^ This in turn will result in biased estimates of the cost-effectiveness of clinical care regimens options.

 Using a micro-costing approach and pooling data from secondary sources, we estimated daily inpatient costs for three levels of care – general wards, high care wards and ICUs. To allow for greater flexibility in the use of our cost estimates, we distinguish between financial and economic costs. Financial cost which includes direct financial outlays are useful for informing budgeting and planning processes while, economic cost represent the opportunity of costs of alternative use of resources and are useful inputs for cost-effectiveness analysis.

 Given variations in the severity of COVID-19 symptoms, the type and quantity of resources vary by hospital levels of care. As expected, our results show that average costs per patient per day increased with the level of care provided, reflecting an increase in the quantity of resources required for the clinical management of less severe through to critical cases of COVID-19. The highest average daily cost was estimated for ICU admissions and the lowest, for general ward-based care. In general wards, the highest cost driver was shown to be consumable costs, with PPE costs, in turn, contributing the highest to total consumable costs. In ICUs, the highest economic costs driver was human resource costs, largely due to higher nursing staff costs, reflecting the intensity of care required for inpatient care within ICUs. Although our cost estimates are specific to South Africa and reflect South African - specific unit prices and COVID-19 clinical care guidelines, we provided a detailed breakdown and description of our cost inputs, thus facilitating the adaptation of our costs estimates to different settings.

 Given the novelty of the disease, there is a dearth of evidence on the costs of clinical management of COVID-19. However, two studies estimating costs in South Africa provide some basis for comparison. In a recent study on the societal economic costs of COVID-19 in five countries including South Africa, Davies et al found similar estimates to our cost estimates for general ward (159 USD) and ICU care (717 USD).^35^ In another study, Mahomed & Mahomed estimated daily ICU costs in South Africa for other conditions.^36^ Although their estimates were higher than ours, Mahomed & Mahomed found that human resource costs was the highest cost driver of total ICUs costs, similar to our findings.^36^ There are a few limitations of our study worth highlighting. First, our cost estimates do not account for some cost items such as costs of training healthcare workers in the clinical management of the novel disease. These costs are likely to be important if health workers have to be redeployed to provide care to COVID-19 patients. Therefore, our study may have underestimated the total costs of clinical management of COVID-19. Second, some important resource quantities including the probability of patients requiring specific therapeutic agents or diagnostic tests and the incidence of complications were largely based on evidence from other countries. Although our final costs estimates were robust to uncertainty in these inputs, variations in disease severity and risk factors across countries may result in variations in these inputs across settings, potentially resulting in biased estimates of our average inpatient care costs. Furthermore, although the identification and measurement of the majority of resource inputs were based on South African-specific guidelines, this represents normative best practices which may differ from real clinical practices, a limitation further exacerbated by evolving clinical guidelines in South Africa as new clinical evidence became available. Therefore, future studies collecting primary data extracted from hospitalised patient records could provide useful insights into real-world quantities of resources used in the management of COVID-19 in South Africa as well as inform heterogeneity of costs.

## Conclusion

 This study provides useful insights into the costs of clinical management of COVID-19 in South Africa, an important input for informing COVID-19 budgets and the efficient allocation of resources for the management or prevention of COVID-19. Decisions to expand various clinical care options or invest in new COVID-19 interventions need to take into account the benefits of alternative use of resources as well as costs and non-financial constraints, such as human resource constraints facing healthcare systems.^36^ Cost estimates presented in this study would be useful for future analysis of the cost-effectiveness of new effective interventions for the clinical management of COVID-19 as well as the cost-effectiveness of COVID-19 preventative interventions.

## Ethical issues

 Ethical approval was not necessary for the study because secondary data was used throughout.

## Competing interests

 Authors declare that they have no competing interests.

## Authors’ contributions

 IE, WM, and HF contributed to the conception and design of the study; all authors contributed to the acquisition and interpretation of data; the first draft of the manuscript was produced by IE, WM, and HF; all authors critically revised the manuscript for intellectual content.

## Funding

 This research was supported by the South African Medical Research Council (23108).

## Endnotes

 [1] We assume that all severe complications from COVID-19 are managed within ICUs.

## Supplementary files

Supplementary file 1 contains Tables S1-S3.

Supplementary file 2 contains Tables S4-S6.

Supplementary file 3 contains Tables S7-S9.

Supplementary file 4 contains Figures S1-S6.
