# Supplementary material for: Inpatient Care Costs of COVID-19 in South Africa’s Public Healthcare System
Source: Int J Health Policy Manag. 2021 Apr 25;11(8):1354–61. doi: 10.34172/ijhpm.2021.24 (PMC9808349; doi:10.34172/ijhpm.2021.24)
Supplement: Supplementary file 1 — contains Tables S1-S3. [file ijhpm-11-1354-s001.pdf]

Supplementary file 1

This material provides a detailed breakdown of the inputs used in estimating cost per patient per day for each resource item, for each level of care – general wards, high care wards (HCW) and intensive care units (ICU).

Diagnostics

Haematology, chest x-rays and biochemistry laboratory testing are assumed to be performed on each admission of a patient with severe or critical COVID-19 disease, and as clinically indicated to monitor for complications, such as acute respiratory distress syndrome, acute kidney injury, acute cardiac injury and septic shock. It was assumed that the SARS CoV-2 Polymerase Chain Reaction (PCR) test would be conducted in general and high care wards, prior to a patient's transfer to ICU, and thus this cost was excluded from ICU. Diagnostics included in this analysis (Table S1) and the frequency with which they are administered were identified from South African and WHO clinical management guidelines, as well as from published studies and expert consultations.[1–5] Unit costs for most diagnostics were obtained from previously published estimates in South Africa and from the South African National Health Laboratory Service (NHLS).[6,7] Unit costs from previous years were inflated to 2020 prices using the Consumer Price Index. [8]

In the probabilistic sensitivity analysis (PSA), average quantity of diagnostic test used per day was varied over a range determined by the frequency of each diagnostic test and the length of hospital stay. For example general and high care wards, the frequency of tests required were varied between daily and once every three days, where the base case required each test every two days. Details of all the ranges used in the PSA are provided in Table S1. To restrict the random draws from 0 to 1, a Beta distribution was fitted to average quantity per day in the PSA.

The length of stay was varied in the PSA, using a lognormal distribution, over a specified range reported by the South African COVID-19 Modelling Consortium. [9] The impact of the length of stay parameter on the final results was also assessed in a scenario analysis, where the lowest and highest value in the range was used as the input.

Therapeutics

Therapeutics for managing hospitalised COVID-19 were categorized into antibiotics/antimicrobials, corticosteroids, anticoagulants and nutritional support (Table S2.) Drugs and quantity requirements included in the analysis were obtained from published studies, clinical guidelines and expert consultations .[3,10–12] Unit prices were obtained from the National Department of Health Master Procurement Catalogue.[13,14] The proportion of patients requiring each therapy was obtained from a review of clinical studies on clinical outcomes of severe or critical COVID-19 patients.[10,15–18] Uncertainty in these proportions were assessed in the PSA, using the confidence intervals provided in the clinical studies (Table S2) to fit Beta distributions to these inputs.

Table S1. Inputs for Diagnostics Costs (2020 USD)

(1) Resource input	Hospital ward applicable	(2) Frequency	(3) Average quantity per day[a] (range)	(4) Unit cost	Cost per patient per day	Source
**Chest X-ray**	General ward	3 over duration of hospitalisation	0.6 (0.3 - 1)	15.05[b]	9.03	(1)[4] (2) [10,19] (3) [10,19] (4) [20]
	High Care and ICUU CPAP	Every two days	0.5 (0.33 - 1)		7.52	
	ICU NIV / IMV	Daily	1 (0.75 - 1)		15.05	
**Full blood count**	General wards, High care wards and ICU CPAP	Every two days	0.5 (0.33 -1)	3.85	1.93	(1) [4] (2) [10] (3) [10] (4) [7]
	ICU NIV / IMV	Daily	1 (0.75 - 1)		3.85	
**Urea**	General wards, High care wards and ICU CPAP	Every two days	0.5 (0.33 -1)	2.02	1.01	(1) [3] (2) [10] (3) [10] (4) [7]
	ICU NIV / IMV	Daily	1 (0.75 - 1)		2.02	
**CRP**	General wards, High care wards and ICU CPAP	Every two days	0.5 (0.33 -1)	4.7	2.49	(1) [2] (2) [10] (3) [10] (4) [7]
	ICU NIV / IMV	Daily	1 (0.75 - 1)		4.97	
**ESR**	General wards, High care wards and ICU CPAP	Every two days	0.5 (0.33 -1)	2.34	1.18	(1) [2] (2) [10] (3) [10]

	ICU NIV / IMV	Daily	1 (0.75 - 1)		2.36	(4) [7]
**Blood gas analysis**	General wards, High care wards and ICU CPAP	Every two days	0.5 (0.33 -1)	4.07	2.03	(1) [3]<br>(2) [19]<br>(3) [19]<br>(4) [6]
	ICU NIV / IMV	Daily	1 (0.75 - 1)		4.07	
**HIV**	General ward	Once per admission	0.04	0.58	0.07	(1) [21]<br>(2) Expert opinion<br>(3) [9]<br>(4) [6]
	High care ward, ICU CPAP / NIV		0.13 (0.08 -0.25)		0.08	
	ICU IMV		0.06 (0.03 – 0.1)		0.04	
**TB Sputum microscopy, culture and susceptibility**	General ward	Once per admission	0.13 (0.08 -0.25)	3.21	0.40	(1) [21]<br>(2) Expert opinion<br>(3) [9]<br>(4) [6]
	High care ward, ICU CPAP / NIV		0.14 (0.08 – 0.33)		0.46	
	ICU IMV		0.06 (0.03 – 0.1)		0.20	
**SARS CoV-2PCR**	General wards	One required for duration of hospitalisation [1]	0.13 (0.08 -0.25)	24.04	3.00	(1) Expert opinion<br>(2) [1]<br>(3)[9]<br>(4) [22]
	High care wards		0.14 (0.08 – 0.33)		3.44	

Note: For most inputs, a different source was used for each component of the input. Thus for each row, the number attached to the column header corresponds to each citation in the source column.

Abbreviations: CPR, C-Reactive Protein; PCR, Polymerase Chain Reaction; ICU: Intensive Care Unit; CPAP: Continuous Positive Airway Pressure; NIV: Non-Invasive Ventilation; IMV: Invasive Mechanical Ventilation

[a]Estimated using average length of stays: 8 (4 - 12) days for general ward; 7 (3 - 12.6) days for HCW and ICU without ventilation; 16 (9.9 – 30.5) days for ICU with invasive mechanical ventilation [9]

[b] The cost obtained from UPFS is specific to chest x-rays, we assume this covers the appropriate number of films required

Table S2. Inputs for Therapeutic Agent Costs (2020 USD)

(1) Resource input	(2) Therapy	(3) Proportion of patients receiving therapy (CI)	(4) Dose	(5) Quantity used per day	(6) Unit cost	Wards applicable	Cost per patient per day	Source
**Antibiotics/ antimicrobials**	Ceftriaxone	72% (56 – 88)	1g IV daily	1	0.37 per 1 g vial	All	1.02	(1) [18] (2) [12 23] (3) [15] (4) [12] (5) [12 23] (6) [13]
	Clarithromycin		500mg 12 hourly	4	0.26 per 250mg dose			
**Corticosteroids**	Dexamethasone	100%[a]	6mg per day	1.5	0.38 per 4mg vial	General wards [b]; HCW and ICU	0.57	(1) [18] (2) [18] (3)[35] (4) [12] (5)[17] (6) [13]
**Anticoagulation**	Enoxaparin	65% (56 – 75)	Regular: 40mg daily	1	2.54 for 40mg injection	All	4.32	(1) [18] (2) [24] (3) [25] (4)[24] (5)[25] (6)[13]
		35% (25 – 44)	High dose: 120mg daily	3				
**Enteral nutrition**	Standard bag with electrolytes	100%	Total Parenteral Nutrition: 1 bag daily	1.00	47.79 per bag [c]	ICU	47.79	(1)[26] (2) [25] (3) [24] (4) [24] (5) [13]

Note: For most inputs, a different source was used for each component of the input. Thus for each row, the number attached to the column header corresponds to each citation in the source column.

Abbreviations: CPAP: Continuous positive airway pressure ventilation; HCW: High care wards; ICU: Intensive care unit.

[a] Based on RECOVERY trial preliminary results, dexamethasone is recommended for all patients receiving respiratory support [18]

[b] Patients receiving supplemental oxygen only [18]

[c] Median price of 3 different options for Adult parenteral nutrition, in absence of estimate for enteral nutrition

Treatment of Complications

Complications resulting from COVID-19, and their incidence, were identified from published studies (Table S3). Complications considered in this analysis include acute cardiac injury, acute liver failure, septic shock and acute kidney injury (Table S3). We assumed that all patients with these complications would receive care in ICU, and thus these costs were only included in estimates of total ICU daily costs. We used the South African Standard Treatment Guidelines to identify treatment regimens and resources required for managing septic shock, acute liver failure and acute cardiac injury.[25] Unit costs for each resource were obtained from the South Africa Master Procurement Catalogue (2020).[13,14] Finally, costs for renal replacement therapy and for acute kidney injury were obtained from the South Africa Uniform Patient Fee Schedule (2019).[20] Treatment costs for acute respiratory distress syndrome are discussed in the following sub-section.

Proportions of patients being affected by each complication (incidence rates) were obtained from a review of clinical studies on outcomes in patients with COVID 19. [15,16,27] We assumed that 50% of septic shock cases would be resolved through providing fluids, while 50% of cases would require vasoconstrictive agents. Similarly, we assumed that 50% of patients with acute cardiac injury would require primary percutaneous coronary intervention (PCI), while 50% would be managed conservatively using medication (fibrinolysis, aspirin and morphine).

In the PSA, the incidence rates of each complication was varied using the confidence intervals presented in the clinical studies, with Beta distributions fitted to these inputs. Where confidence intervals were not available from the clinical study (as was the case with the incidence rate for pneumothorax only), the rate was varied by +/-50%.

Table S3. Inputs for Treatment of Complications Costs (2020 USD)

(1)<br>Complication	(2)<br>Incidence of complication [CI]	(3)<br>Therapy	(4)<br>Proportion requiring therapy	(5)<br>Quantity used per day [25]	(6)<br>Cost per dose	Cost per patient per day	Source
**Septic shock**	19.7%<br>[4%-28%]	Fluids (sodium chloride 0.9%)	50%	500ml IV over 30 minutes	0.26	14.76 [a]	(1) [28]<br>(2) [28]<br>(3) [25]<br>(4) Assumption<br>(5) [25]<br>(6) [13]
		Vasoconstrictive agents (epinephrine)	50%	576ml per day	149.93		
**Acute kidney injury (end stage, requiring dialysis)**	6.8%<br>[1% – 17%]	Renal replacement therapy	100%	24 hours of therapy per day	126.57	8.61 [a]	(1) [29]<br>(2) [29]<br>(3) [25]<br>(4) Assumption<br>(5) Assumption<br>(6) [20]
**Acute liver failure**	26.5%<br>[9% – 41%]	Lactulose	100%	10–30 mL 8 hourly	5.25	0.17 [a]	(1)[30]<br>(2) [30]<br>(3) [25]<br>(4) Assumption<br>(5) [25]<br>(6) [13]
**Acute cardiac injury**	20%<br>[16% - 24%]	Primary percutaneous coronary intervention (PCI)	50%	Once-off intervention	4367.53	62.55 [b]	(1) [31]<br>(2) [31]<br>(3) [25]<br>(4) Assumption<br>(5) Assumption<br>(6) [32]
						27.64 [c]	
		Fibrinolysis (streptokinase)	50%	1.5MU IV, once-off	2.94	6.84 [b]	(1) [31]<br>(2) [31]

		Aspirin		150mg - 300 mg	0.009		(3) [25]<br>(4) Assumption
		Morphine		10mg	0.20*	2.94 [c]	(5) [25]<br>(6) [13]
**Pneumothorax**	0.66%<br>[0.33% – 0.99%]	Intercostal drain	100%	One per admission	4.83	0.004 [b] / 0.002 [c]	(1) [33]<br>(2) [33]<br>(3) [25]<br>(4) Assumption<br>(5) Assumption<br>(6) [34]

Note: For most inputs, a different source was used for each component of the input. Thus for each row, the number attached to the column header corresponds to each citation in the source column.

[a] All ICU patients

[b] ICU patients on CPAP or NIV

[c] ICU patients on invasive mechanical ventilation

References

1. National Department of Health South Africa,national Institute of communicable Disease South Africa. Clinical management of suspected or confirmed COVID-19 disease Version 4. 2020 May. Report No.: Version 4.
2. Yang X, Yu Y, Xu J, Shu H, Xia J, Liu H, et al. Clinical course and outcomes of critically ill patients with SARS-CoV-2 pneumonia in Wuhan, China: a single-centered, retrospective, observational study. Lancet Respir Med. 2020 May 1;8(5):475–81.
3. Li T, Lu H, Zhang W. Clinical observation and management of COVID-19 patients. Emerg Microbes Infect. 2020 Jan 1;9(1):687–90.
4. Zhou F, Yu T, Du R, Fan G, Liu Y, Liu Z, et al. Clinical course and risk factors for mortality of adult inpatients with COVID-19 in Wuhan, China: a retrospective cohort study. The Lancet. 2020 Mar 28;395(10229):1054–62.
5. World Health Organization. Clinical management of severe acute respiratory infection (SARI) when COVID-19 disease is suspected -Interim Guidance [Internet]. 2020. Available from: file:///C:/Users/Noel/Downloads/WHO%20Clinical%20management%20of%20severe%20acute%20respiratory%20infection%20when%20COVID19%20is%20suspected.pdf
6. National Health Laboratory Service. NHLS State Price List 2013.
7. Tempia S, Moyes J, Cohen AL, Walaza S, Edoka I, McMorrow ML, et al. Health and economic burden of influenza-associated illness in South Africa, 2013-2015. Influenza Other Respir Viruses. 2019/06/11 ed. 2019 Sep;13(5):484–95.
8. Statistics South Africa. Consumer Price Index, April 2020. Pretoria: Statistics South Africa, Government of the Republic of South Africa; 2020.
9. MASHA, HE2RO, SACEMA. Estimating cases for COVID-19 in South Africa: Assessment of alternative scenarios. National Institute for Communiable Diseases; 2020 Sep.
10. Yang X, Yu Y, Xu J, Shu H, Xia J, Liu H, et al. Clinical course and outcomes of critically ill patients with SARS-CoV-2 pneumonia in Wuhan, China: a single-centered, retrospective, observational study. Lancet Respir Med. 2020 May 1;8(5):475–81.
11. Nicastri E, Petrosillo N, Ascoli Bartoli T, Lepore L, Mondi A, Palmieri F, et al. National Institute for the Infectious Diseases “L. Spallanzani”, IRCCS. Recommendations for COVID-19 clinical management. Infect Dis Rep. 2020 Mar 16;12(1):8543–8543.
12. Wasserman Sean, Boyles Tom, Mendelson Mark. A pocket guide to prescribing antibiotics for adults in South Africa , 2015. South African Antibiotic Stewardship Programme;
13. Master Procurement Catalogue 2020 [Internet]. 2020. Available from: http://www.health.gov.za/index.php/component/phocadownload/category/196
14. Master Procurement Catalogue 2019. 2019.
15. Sanders JM, Monogue ML, Jodlowski TZ, Cutrell JB. Pharmacologic Treatments for Coronavirus Disease 2019 (COVID-19): A Review. JAMA. 2020 May 12;323(18):1824–36.
16. Hirsch JS, Ng JH, Ross DW, Sharma P, Shah HH, Barnett RL, et al. Acute kidney injury in patients hospitalized with COVID-19. Kidney Int. 2020 Jul 1;98(1):209–18.
17. RECOVERY Collaborative Group, Horby P, Lim WS, Emberson JR, Mafham M, Bell JL, et al. Dexamethasone in Hospitalized Patients with Covid-19 - Preliminary Report. N Engl J Med. 2020 Jul 17;NEJMoa2021436.
18. National Institutes of Health. COVID-19 Treatment Guidelines [Internet]. [cited 2020 Aug 24]. Available from: https://www.covid19treatmentguidelines.nih.gov/immune-based-therapy/immunomodulators/corticosteroids/

19. Li T. Diagnosis and clinical management of severe acute respiratory syndrome Coronavirus 2 (SARS-CoV-2) infection: an operational recommendation of Peking Union Medical College Hospital (V2.0). Emerg Microbes Infect. 2020 Jan 1;9(1):582–5.
20. National Department of Health South Africa. Uniform Patient Fee Schedule 2020 [Internet]. National Department of Health South Africa; 2020 [cited 2020 Jul 20]. Available from: http://www.health.gov.za/index.php/uniform-patient-fee-schedule/category/652-twenty
21. Davies NG, Sweeney S, Torres-Rueda S, Bozzani F, Kitson N, Barasa E, et al. The impact of Coronavirus disease 2019 (COVID-19) on health systems and household resources in Africa and South Asia (pre-print). medRxiv. 2020 Jan 1;2020.05.06.20092734.
22. COVID-19 Cost Working Group. National COVID-19 Budget Model South Africa. 2020 Apr.
23. Ministerial Advisory Committee on Antimicrobial Resistance. Guidelines on Implementation of the Antimicrobial Strategy in South Africa: One Health Approach & Governance June 2017.
24. Gutierrez Christina, Franco-Martinez Crystal, Gonzales Jose, Foster Dana. Guidelines for Anticoagulation in Hospitalized COVID-19 Patients ≥ 18 Years of Age. University Health System;
25. National Department of Health, Essential Drugs Program. Standard Treatment Guidelines and Essential Medicines List 5th Edition.2019. Pretoria South Africa: National Department Of Health;
26. Thibault R, Seguin P, Tamion F, Pichard C, Singer P. Nutrition of the COVID-19 patient in the intensive care unit (ICU): a practical guidance. Crit Care Lond Engl. 2020 Jul 19;24(1):447–447.
27. BMJ Best Practice. Coronavirus disease 2019 (COVID-19)-Complications [Internet]. BMJ Best Practice. 2020 [cited 2020 Jul 15]. Available from: https://bestpractice.bmj.com/topics/en-us/3000168/complications
28. Lakoh S, Jiba D, Baldeh M, Vandy A, BENYA H, Lado M, et al. Sepsis and septic shock in COVID-19: a scoping review of the research data. 2020.
29. Kunutsor S, Laukkanen J. Hepatic manifestations and complications of COVID-19: A systematic review and meta-analysis. J Infect. 2020 Jun 1;
30. Sharma A, Jaiswal P, Kerakhan Y, Saravanan L, Murtaza Z, Zergham A, et al. Liver disease and outcomes among COVID-19 hospitalized patients – A systematic review and meta-analysis. Ann Hepatol [Internet]. 2020 Oct 16; Available from: http://www.sciencedirect.com/science/article/pii/S1665268120301885
31. Prasitlumkum N, Chokesuwattanaskul R, Thongprayoon C, Bathini T, Vallabhajosyula S, Cheungpasitporn W. Incidence of Myocardial Injury in COVID-19-Infected Patients: A Systematic Review and Meta-Analysis. Dis Basel Switz [Internet]. 2020 Oct;8(4). Available from: https://doi.org/10.3390/diseases8040040
32. Stassen W, Wallis L, Vincent-Lambert C, Castrén M, Kurland L. Percutaneous coronary intervention still not accessible for many South Africans. Afr J Emerg Med. 2017 Apr 1;7.
33. Zantah M, Dominguez Castillo E, Townsend R, Dikengil F, Criner GJ. Pneumothorax in COVID-19 disease- incidence and clinical characteristics. Respir Res. 2020 Sep 16;21(1):236.
34. Afrimedics. Drainage system closed wound 3mm [Internet]. Afrimedics. [cited 2020 Dec 1]. Available from: https://afrimedics.co.za/product/drainage-system-closed-wound-3mm/
