# Supplementary material for: Inpatient Care Costs of COVID-19 in South Africa’s Public Healthcare System
Source: Int J Health Policy Manag. 2021 Apr 25;11(8):1354–61. doi: 10.34172/ijhpm.2021.24 (PMC9808349; doi:10.34172/ijhpm.2021.24)
Supplement: Supplementary file 2 — contains Tables S4-S6. [file ijhpm-11-1354-s002.pdf]

Supplementary file 2

Respiratory Support

As specified in the main paper, different proportions of patients admitted to each ward are assumed to be administered specific respiratory support therapy. In general wards, patients may or may not require respiratory support. For those requiring respiratory support, supplemental oxygen is administered via a face mask (50% of patients) or nasal cannula (50% of patients). In high care wards, patients are assumed to receive high flow nasal oxygen (HFNO). In intensive care units, patients are assumed to receive respiratory support in the form of continuous positive airway pressure (CPAP), bi-phasic non-invasive ventilation (NIV) or mechanical invasive ventilation.

We assumed that oxygen was supplied using oxygen concentrators in all ward types, while backup oxygen tanks are supplied for high care wards and intensive care units. These items are "capital equipment" and are presented in Table S4.

Oxygen volumes per minute required for each type of respiratory support were obtained from the South African guidelines for the clinical management of suspected or confirmed COVID-19 disease, consultation with clinical experts, and other published clinical recommendations (Table S4).[1,2] Oxygen volume, expressed in litres per minute was converted to daily oxygen requirements expressed in kilograms (kg). This was applied to unit cost of oxygen per kg to obtain cost per patient per day for each type of respiratory support. The unit cost was based on the current public-sector hospital supplies tender from the South Africa National COVID-19 Budget Model. [3]

Resource items and quantities of consumables required for ventilator respiratory support were obtained from the WHO COVID-19 Essential Supplies Forecasting Tool (ESFT), [4] while unit cost estimates were derived from the KwaZulu-Natal Department of Health procurement documents, the National COVID Budget Model as well as listed prices in the private sector. Unit costs for high flow nasal oxygen (HFNO) were obtained from the South African National COVID-19 Budget Model.

Unit costs of consumables for supplemental oxygen therapy in general wards were obtained from KwaZulu-Natal Department of Health procurement documents.[5] These consumables include nasal cannula and an adjustable Venturi face mask where we assumed that 50% of patients would require a face mask, and 50% of patients would require nasal cannula (Table S4).

The cost of the line items that are required only once per admission were divided by average length of stay to estimate the average cost per patient per day. In the PSA, this average length

of stay was varied using a lognormal distribution.[6] The impact of the length of stay on the final results was also assessed in the scenario analysis.

Table S1. Inputs for Respiratory Support Costs (2020 USD)

(1) Resource input	(2) Quantity	Ward Applicable	(3) Unit cost	Cost per patient per day	Source
**Oxygen flow**	10L/min = 20.58kg per day	General ward	0.78 per kg	16.12	(1) [4]<br>(2) [7]<br>(3) [3]
	35L/min = 72.02kg per day	High care ward		56.43	(1) [4]<br>(2) [2]<br>(3) [3]
	30L/min = 61.73kg per day	ICU		48.37	(1)[4]<br>(2) [3]<br>(3) [3]
**Oxygen face mask**	One per admission [a]	General ward	0.83	0.05	(1) [4]<br>(2) Expert opinion<br>(3) [8]
**Nasal cannula**	One per admission [a]	General ward	0.28	0.02	(1) [4]<br>(2) Expert opinion<br>(3) [8]
**High flow machine consumables**	One per admission	High care ward	120.36	17.24	(1) [4]<br>(2) Expert opinion<br>(3)[3]
**CPAP mask (anaesthetic mask, cushioned)**	One per admission	ICU: CPAP	1.82	0.26	(1) [4]<br>(2) Assumption<br>(3) [8]

**NIV mask (anaesthetic mask, cushioned)**	One per admission	ICU: NIV	1.64	0.24	(1)[4]<br>(2) Assumption<br>(3) [8]
**Suction catheters**	1[b]	ICU: CPAP/NIV	0.23	0.23	(1) [4]<br>(2)Assumption<br>(3) [9]
	3[c]	ICU: IMV		0.68	
**Oropharyngeal airway**	One per admission	ICU: IMV	0.18	0.01	(1) [4]<br>(2) Assumption<br>(3) [8]
**Endotracheal tube**	One per admission	ICU: IMV	1.15	0.07	(1) [4]<br>(2)Assumption<br>(3) [8]

Note: For most inputs, a different source was used for each component of the input. Thus for each row, the number attached to the column header corresponds to the number attached to each citation in the source column.

[a] Assume 50% of patients require a face mask, and 50% of patients require nasal cannula.

[b]Assume 1 per day for assessment suction for patients on NIV

[c] Assume 8 hourly suction for intubated patients

Personal Protective Equipment (PPE)

Basic PPE needed by each clinical staff include medical mask, gown, non-sterile gloves and eye protection goggles. N95 masks were assumed to be worn only in wards where aerosol generating procedures (AGPs) were performed i.e. in high care wards and ICU. [10]

PPE types, unit costs and quantity required per day are presented in Table S5. We considered only the costs of PPE worn by clinical staff providing direct care to COVID-19 patients, as the National Department of Health Infection Prevention and Control Guidelines for COVID-19 do not recommend that facemasks be worn by patients in dedicated COVID-19 ward were all staff are wearing PPE. [10] The list of PPE items required as well as their quantities were obtained from these the National Department of Health Infection Prevention and Control Guidelines for COVID-19. [10]

The National Department of Health Infection Prevention and Control Guidelines for COVID-19 calculated quantities of each PPE item that would be required, based on an assumption of a capacity of 30 patients in each ward or unit. This means that the staff on each unit would not need to change PPE (aside from gloves) in between treating different patients. The guidelines calculated quantity of PPE required per hospital ward per day, based on the average number of staff in each ward (general ward, HCW and ICU) and the assumed staff-to-patient ratio. Therefore, we calculated PPE quantities required per patient per day based on the quantity of PPE required per ward per day, divided by the number of patients in each ward. In the scenario analysis, the capacity of each unit was varied from a lower bound of 20 patients to an upper bound of 40 patients, in order to assess the impact of this assumption on the final results.

The quantities of PPE estimated this way were applied to the unit costs of each item to estimate the cost per patient per day for PPE. Unit costs for PPE were obtained from the South African National Treasury COVID-19 PPE price list .[11]

Table S2. Inputs for Personal Protective Equipment Costs (2020 USD)

(1) Resource input	(2) Unit cost per item	(3) Quantity per patient per day (range)	Ward	Cost per patient per day	Source
**Non-sterile gloves (in pairs)**	0.11	24 (18 - 36)	General ward	2.60	(1)[11]
		36 (27 54)	High care ward	3.90	(2) [11]
		48 (36 – 72)	Intensive care	5.20	(3) Assumption
**Goggles** [a]	6.04	0.33 (0.25 0.5)	General ward	2.01	(1)[11]
		0.5 (0.38 – 0.75)	High care ward	3.02	(2) [11]
		0.67 (0.5 - 1)	Intensive care	4.03	(3) Assumption
**Visors** [a]	6.50	0.33 (0.25 – 0.5)	General ward	2.17	(1)[11]
		0.5 (0.38 – 0.75)	High care ward	3.25	(2) [11]
		0.67 (0.5 1)	Intensive care	4.33	(3) Assumption
**Plastic aprons**	0.18	2 (1.5 - 3)	General ward	0.36	(1)[11]
		3 (2.25 - 4.5)	High care ward	0.54	(2) [11]
		4 (3 6)	Intensive care	0.71	(3) Assumption
**Cotton gowns with apron**	6.82	2 (1.5 – 3)	General ward	13.65	(1)[11]
		3 (2.25 4.5)	High care ward	20.47	(2) [11]
		4 (3 6)	Intensive care	27.30	(3) Assumption
**Surgical face masks**	1.08	2 (1.5 - 3)	General ward	2.17	(1)[11]
		3 (2.25 4.5)	High care ward	3.25	(2) [11]
		4 (3 6)	Intensive care	4.33	(3) Assumption

**-N95 respirator (for AGP only)**	2.27	1 (0.75 - 1.5)	General ward	2.27	(1)[11]
		1.5 (1.13 2.25)	High care ward	3.41	(2) [11]
		2 (1.5 3)	Intensive care ward	4.55	(3) Assumption
**Water resistant gowns (for AGP only)**	8.12	1 (0.75 - 1.5)	General ward	8.12	(1)[11]
		1.5 (1.13 2.25)	High care ward	12.19	(2) [11]
		2 (1.5 3)	Intensive care ward	16.25	(3) Assumption

Note: For most inputs, a different source was used for each component of the input. Thus for each row, the number attached to the column header corresponds to the number attached to each citation in the source column.

Abbreviations: AGP, Aerosol generating procedures**.**

[a] Assumption: Either goggles or visors are used. 50% of health care workers use goggles, 50% use visors.

Hygiene Infection and Control

Resource type, unit costs and quantity required per day for hygiene and infection control purposes are presented in Table S6. Resources used for hygiene infection and control include 70% alcohol detergent disinfectant (for disinfecting equipment), 0.5% sodium hypochlorite disinfectant (for disinfecting surfaces), as well as alcohol-based hand rub, liquid hand soap, and paper towels for hand hygiene. Information on hygiene infection and control requirements and their quantities was obtained from the National Department of Health Infection Prevention and Control Guidelines for COVID-19.[10] Unit costs for hygiene infection and control resources were obtained from the South African National Treasury COVID-19 PPE price list, government commodity tender documents and retail advertisements. [11] Similar to the scenario analysis conducted for PPE quantities required, we varied the number of patients per unit that each hygiene and infection control line item was attributed to, from a lower bound of 20 patients to an upper bound of 40 patients per unit (where the base case assumed 30 patients).

Table S3. Inputs for Hygiene Infection and Control Costs (2020 USD)

(1)<br>Resource input	(2)<br>Unit cost	(3)<br>Quantity, per patient per day (range)	Ward	Cost per patient per day	Sources
**Hand rub**	11.05 per litre	0.1L (0.08 - 0.15)	General	1.10	(1)[11]<br>(2) [11]<br>(3) Assumption
		0.15L (0.11 - 0.23)	High care	1.66	
		0.2L (0.15 - 0.3)	Intensive care	2.21	
**Liquid hand soap**	1.23 per litre	0.05L (0.08 - 0.08)	General	0.06	(1)[11]<br>(2) [12]<br>(3) Expert opinion
		0.08L (0.06 - 0.11)	High care	0.09	
		0.1L (0.075 - 0.15)	Intensive care	0.12	
**Paper towels**	0.01 per hand towel	50 (37.5 - 75)	General	0.58	(1) [10]<br>(2) [13]<br>(3) Expert opinion
		75 (56.25 - 112.5)	High care	0.87	
		100 (75 - 150)	Intensive care	1.16	
**70% alcohol disinfectant**	11.05 per litre	0.067L (0.05 - 0.1)	General	0.74	(1)[11]<br>(2) [11]<br>(3) Assumption
		0.1L (0.075 - 0.15)	High care	1.10	
		0.13 (0.1 – 0.2)	Intensive care	1.47	
**0.5% sodium hypochlorite (for surface disinfection)**	0.72 per litre	0.33L (0.25 - 0.5)	General	0.24	(1)[11]<br>(2) [12]<br>(3) Expert opinion
		0.42L (0.31 - 0.63)	High care	0.30	
		0.5L (0.38 - 0.75)	Intensive care	0.36	

Note: For most inputs, a different source was used for each component of the input. Thus for each row, the number attached to the column header corresponds to each citation in the source column.

References

1. National Department of Health South Africa,national Institute of communicable Disease South Africa. Clinical management of suspected or confirmed COVID-19 disease Version 4. 2020 May. Report No.: Version 4.
2. Cascella M, Rajnik M, Cuomo A, Dulebohn SC, Di Napoli R. Features, Evaluation, and Treatment of Coronavirus (COVID-19) [Internet]. Treasure Island (FL): StatPearls Publishing; 2020. Available from: https://www.ncbi.nlm.nih.gov/books/NBK554776/
3. COVID-19 Cost Working Group. National COVID-19 Budget Model South Africa. 2020 Apr.
4. World Health Organization. WHO COVID-19 Essential Supplies Forecasting Tool (ESFT) [Internet]. Available from: https://www.who.int/publications/m/item/covid-19-essential-supplies-forecasting-tool
5. Department of Health Province of Kwazulu Natal. ZNB 6685/1/2018-H: Supply and delivery of respiratory aids and related items for various institutionsfor a period of 3 years [Internet]. 2020. Available from: http://www.kznhealth.gov.za/Tenders/Awarded/2020/ZNB6685.12018-H.pdf
6. MASHA, HE2RO, SACEMA. Estimating cases for COVID-19 in South Africa: Assessment of alternative scenarios. National Institute for Communiable Diseases; 2020 Sep.
7. Dondorp AM, Hayat M, Aryal D, Beane A, Schultz MJ. Respiratory Support in COVID-19 Patients, with a Focus on Resource-Limited Settings. Am J Trop Med Hyg. 2020 Jun;102(6):1191–7.
8. Department of Health Province of Kwazulu Natal. ZNB 6685/1/2016-H: Supply and delivery of respiratory aids :Part one for various institutions [Internet]. 2017. Available from: http://www.kznhealth.gov.za/Tenders/Awarded/2017/ZNB6685.1.2016-H.pdf
9. The Paramedic Shop. Suction Catheters with Control [Internet]. 2020 [cited 2020 May 14]. Available from: https://www.paramedicshop.co.za/products/suction-catheters-with-control
10. National Department of Health,South Africa. National Department of Health Infection Prevention and Control Guidelines for Covid 19 [Internet]. National Department of Health; 2020 May [cited 2020 Apr 15]. Report No.: Version 2. Available from: https://www.nicd.ac.za/wp-content/uploads/2020/05/ipc-guidelines-covid-19-version-2-21-may-2020.pdf
11. South African National Treasury. COVID-19 Personal Protective Equipment Price List [Internet]. South African National Treasury; 2020 [cited 2020 Apr 29]. Available from: http://www.treasury.gov.za/comm_media/press/2020/Annexure%20A%20-%20PPE%20Price%20List.pdf
12. 3pin. Hand soap and sanitiser consumables [Internet]. Washroom Equipment and consumables. 2020 [cited 2020 Apr 14]. Available from: http://www.3pin.co.za/ecommerce/category-browse-subcategory?usca%5fp=t&category%5fid=21&subcategory%5fid=197&subsubcategory%5fid=0&how%5fmany=25&start=0
13. 3Pin.co.za. Kimberly-Clark Folded Paper Towels [Internet]. 2020. Available from: Kimberly-clark, http://www.3pin.co.za/ecommerce/product-search?search_text=paper+towels&sa=Search
