# Supplementary material for: Inpatient Care Costs of COVID-19 in South Africa’s Public Healthcare System
Source: Int J Health Policy Manag. 2021 Apr 25;11(8):1354–61. doi: 10.34172/ijhpm.2021.24 (PMC9808349; doi:10.34172/ijhpm.2021.24)
Supplement: Supplementary file 3 — contains Tables S7-S9. [file ijhpm-11-1354-s003.pdf]

Supplementary file 3

Capital Equipment

We identified hospital equipment and linen requirements through reviewing clinical management guidelines and published literature. [1–3] These items, quantity per patient, their unit costs and corresponding annualised costs are presented in Table S7. Unit costs for capital equipment were obtained from government commodity tender documents and retail advertisements.[4–10] (Table S7). In general wards, we assumed that nurses would observe and record patients' vital signs twice per day, and thus 2 vital sign monitoring machines would be shared amongst 50 patients on a ward. We assumed that patients in high care and intensive care would require continuous monitoring. Quantity of linen used per patient per day was obtained through expert consultations (Table S7).

We assumed that each hospital bed would be occupied for two-thirds of the year, in order to apportion daily economic costs to each patient – this assumption does not apply to the financial cost analysis, where the unit price of each equipment was divided by the total number of days in a year (365 days) to estimate daily financial costs. In a scenario analysis, we assessed the impact of this assumption (number of 'bed days' per year) on total economic costs, using a lower bound of a one-third occupancy rate and an upper bound of 100% occupancy.

Table S1. Inputs for Capital Equipment Costs (2020 USD)

(1)<br>Resource input	Ward/ patient population	(2)<br>Quantity, per patient per day	(3)<br>Unit cost	Annualised costs	Economic cost per patient per day [financial cost]	Sources
**Infusion pump**	All wards	1	897.87	116.28	0.48 [0.25]	(1) [11]<br>(2) Expert opinion<br>(3) [5]
**Patient monitor**	General ward	0.04 [a]	1 992.70	258.06	0.03 [0.01]	(1) [11]<br>(2) Expert opinion<br>(3) [6]
	High care ward	0.083 [b]			0.09 [0.05]	
	ICU	1			1.06 [0.55]	
**Ventilator**	ICU: CPAP	1	9 660.80	1 251.12	5.14 [2.65]	(1) [11]<br>(2)Assumption<br>(3) [4]
	ICU: NIV	1	12 945.12	1 676.45	6.89 [3.55]	
	ICU: IMV	1	26 991.34	3 495.50	14.37 [7.39]	
**High flow machine**	High care ward	1	3 716.34	487.11	2.00 [1.03]	(1) [11]<br>(2) Expert opinion<br>(3) [4]
**Oxygen Concentrator**	All patients who receive respiratory support	1	2 018.26	261.37	1.07 [0.55]	(1) [11]<br>(2) Expert opinion<br>(3) [7]
**Suction pump**	ICU	1	210.63	27.28	0.11 [0.06]	(1) [11]<br>(2) Expert Opinion<br>(3) [8]
**Backup oxygen cylinder**	All patients who receive respiratory support	1	283.53	36.72	0.15 [0.08]	(1) [11]<br>(2)Expert Opinion<br>(3) [9]
**Ambu-bag (self-inflating bag)**	ICU	1	15.72	3.63	0.01[0.01]	(1) [11]<br>(2) Expert Opinion<br>(3) [10]
**Laryngoscope**	ICU: intubated patients only	0.38 [c]	209.75	48.44	0.20 [0.11]	(1) [11]<br>(2) [11,12]

						(3) [13]
**Bed: Manual hospital bed plus mattress**	All	1	620.23	80.33	0.33 [0.17]	(1) [11]<br>(2) Expert Opinion<br>(3) [4]
**Blanket**	All	1	21.24	4.91	0.02 [0.01]	(1) [1]<br>(3) [1]
**Draw sheet**	All	5 [d]	10.23	11.82	0.05 [0.03]	(1) [1]<br>(3) [1]
**Bed sheet**	All	5 [d]	10.23	11.82	0.05 [0.03]	(1) [1]<br>(3) [1]
**Mattress Protector**	All	5 [d]	9.03	10.45	0.05 [0.03]	(1) [1]<br>(3) [1]
**Blanket cover**	All	5 [d]	10.23	11.82	0.05 [0.03]	(1) [1]<br>(3) [1]
**Patient suits**	All	5 [d]	19.86	22.94	0.11 [0.06]	(1) [1]<br>(3) [1]
**Pillows**	All	2	7.22	3.34	0.02 [0.01]	(1) [1]<br>(3) [3]
**Pillow covers**	All	5 [d]	3.41	3.94	0.01 [0.01]	(1) [1]-<br>(3) [2]

Note: For most inputs, a different source was used for each component of the input. Thus for each row, the number attached to the column header corresponds to each citation in the source column.

[a] 2 monitors shared amongst the ward for nurses to take twice daily vitals

[b] Each patient requires 3 hourly observations for approximately 15 minutes, thus 2/24 hours each day

[c] 3 required for unit, thus divided by number of patients in the unit, and only used once during ICU admission

[d] Multiple required per bed space to rotate for laundry

Human Resources

Detailed inputs for estimating cost per patient per day for each cadre are displayed in Table S8. Hourly wage of each cadre of staff was estimated using annual salary, number of hours worked per year, overtime and benefits pay. The value of overtime pay was based on expert opinion and was estimated to be 40% the annual salary of junior staff and 20% the annual salary of senior staff. Benefits are included in the annual salary estimates of staff members included in the Occupational-Specific Dispensation (OSD) salary scales. Administrative staff and cleaning staff are not included in the OSD scales, and these staff receive benefits amounting to 37% of their annual salary (expert consultation).

Staff-to-patient ratios for each staff cadre were determined by dividing the estimated number of patients on each ward (50 patients in general wards, 25 patients in high care wards and 12 patients in ICU) by the number of staff expected to cover each ward (Table S8). In the case of nurses, the staff-to-patient ratio was obtained from the Guidelines for Intensive Care in South Africa, and through expert consultation.[14]

In a scenario analysis, staff-to-patient ratio for each cadre was varied by +/-50% to determine the impact of these assumptions on cost per patient day. The ranges of staff-to-patient ratio used in this sensitivity analysis are presented in Table S8.

Table S2. Inputs for Human Resource Costs (2020 USD)

(1) Type of Human Resource	Ward	(2) Staff-to-patient ratio	(3) Annual salary	(4) Overtime	(5) Benefits	(6) Hourly wage	Cost per patient per day	Source
**Infection prevention control officers**	All wards	1:100 (1:50 – 1:150)	24 717.93	-	-	14.37	1.15	(1) [15]<br>(2) Assumption<br>(3) [16]<br>(6) [16], Expert opinion
**Critical care nurses**	Intensive care Unit	1:2 (1:1 – 1:3)	28 791.00	-	10 652.64	16.74	200.87	(1) [15]<br>(2) Assumption<br>(3) [16]<br>(5)[16]<br>(6) [16],Expert opinion
**Professional nurse**	General ward	1: 18 (1:9 – 1:27)	21 201.65	-	-	12.33	8.22	(1) [14]<br>(2) Expert opinion<br>(3) [16]<br>(6) [16],Expert opinion
	High care ward	1:3 (1:1 – 1:5)					49.31	
	Intensive care unit	1:2 (1:1 – 1:3)					147.92	
**Staff nurses**	General ward	1:18 (1:9 – 1:27)	13 658.467	-	-	7.95	5.29	(1) [14]<br>(2) Expert opinion<br>(3) [16]<br>(6) [16],Expert opinion
	High care ward	1:3 (1:1 – 1:5)					31.76	
	Intensive care unit	1:2 (1:1 – 1:3)					95.29	
**Nursing Assistant**	General ward	1:10 (1:5 – 1:15)	10 468.79	-	-	6.08	14.61	(1) Expert opinion<br>(2) Expert opinion<br>(3) [16]<br>(6) [16],Expert opinion

**Consultant (internal medicine)** [a,b]	General ward and high care ward	1:100 (1:50 – 1:150)	91 676.98	18 335.40	-	63.96	5.12	(1) [14]<br>(2)Expert opinion<br>(3) [16]<br>(4) [16],Expert opinion<br>(6) [16],Expert opinion
	Intensive care Unit	1:12 (1:6 – 1:18)					3.41	
**Intensivist – team leader** [a]	Intensive care Unit	1:12 (1:6 – 1:18)	114 617.63	22 923.50	-	79.97	4.26	(1) [14]<br>(2) Expert opinion<br>(3) [16]<br>(4) [16],Expert opinion<br>(6) [16],Expert opinion
**Cardiovascular specialist** [a]	Intensive care Unit	1:12 (1:6 – 1:18)	91 676.98	18 335.40	-	63.96	0.56	(1) [17]<br>(2) Expert opinion<br>(3) [16]<br>(4) [16],Expert opinion<br>(6) [16],Expert opinion
**Pulmonologists**	Intensive care unit	1:20 (1:10 – 1:30)	91 676.9	18 335.40	-	63.96	2.13	(1) [14]<br>(2) Expert opinion<br>(3) [16]<br>(4) [16],Expert opinion<br>(6) [16],Expert opinion
**Internal medicine physician (registrar)**	General ward	1:25 (1:13 – 1:38)	50 538.92	20 215.58	-	41.14	13.16	(1) [14]<br>(2) Expert opinion<br>(3) [16]<br>(4) [16],Expert opinion<br>(6) [16],Expert opinion
	High care ward	1:20 (1:10 – 1:30)					16.45	
	Intensive care unit	1:12 (1:6 – 1:18)					27.42	
**Administrative staff**	General ward	1:50 (1:25 – 1:75)	9 490.30	-	3 511.40	5.52	0.88	(1) [14]

	High care ward	1:20<br>(1:10 – 1:30)					3.02	(2) [14]<br>(3) [16]<br>(4) [16],Expert opinion<br>(6) [16],Expert opinion
	Intensive care unit	1:12 (1:6 – 1:18)					3.68	
**Respiratory physiotherapists** [c]	High care ward	1:20<br>(1:10 – 1:30)	24 717.93	-	-	14.37	5.75	(1) [14]<br>(2) Expert opinion<br>(3) [16]<br>(4) [16],Expert opinion<br>(6) [16],Expert opinion
**Radiographer c**	High care ward	1:100<br>(1:50 – 1:150)	24 717.93	-	-	14.37	1.15	(1) [14]<br>(2) [14]<br>(3) [16]<br>(4) [16],Expert opinion<br>(6) [16],Expert opinion
	Intensive care unit	1:12<br>(1:6 – 1:18)		9 887.20		20.12	3.22	
**Social worker**	High care wards	1:100<br>(1:50 - 1:150)	23 470	9 388.17	-	19.10	1.09	(1) Expert opinion<br>(2) [14]<br>(3) [16]<br>(4) [16]<br>(6) [16],
	Intensive care unit						3.06	
**Medical technologists**	High care ward	1:100<br>(1:50 – 1:150)	24 717.93	-	-	14.37	1.15	(1) Expert opinion<br>(2) [14]<br>(3) [16]<br>(4) [16],Expert opinion<br>(6) [16],Expert opinion
	Intensive care unit	1:50[c]<br>(1:25 – 1:75)		9 887.20		20.12	3.22	

**Nutritionist**	Intensive care unit	1:50 (1:25 – 1:75)	24 717.93	-	-	14.37	2.30	(1) [14]<br>(2) Expert opinion<br>(3) [16]<br>(4) [16],Expert opinion<br>(6) [16],Expert opinion
**Clinical pharmacist**	High Dependency	1:100 (1:50 – 1:150)	58 221.98	-	-	33.85		(1) [14]<br>(2) [14]<br>(3) [16]<br>(4) [16],Expert opinion<br>(6) [16],Expert opinion
	Intensive care unit	1:50 (1:25 – 1:75)						
**Cleaning staff**	All wards	1:50 (1:25 – 1:75)	6 697.94	-	2 478.27	5.34	0.85	(1) [14]<br>(2) [14]<br>(3) [16]<br>(4) [16]<br>(6) [16]

Note: For most inputs, a different source was used for each component of the input. Thus for each row, the number attached to the column header corresponds to each citation in the source column.

[a] One consultant leading medical team on ICU, with the role shared between Intensivist, anaesthetist consultant and cardiovascular specialist

[b] In the general ward, twenty-four-hour consultant availability, covering HDU and the wards (approximately 100 pts)

[c] Working 8 hours/day full-time, providing on-call cover for 16 hours of the day to ICU only

Overhead costs

Table S9 presents the ward overhead costs. These were obtained from the facility fees laid out in the Uniform Patient Fee Schedule (2020). The UPFS reports fees for 3 different levels of health care facilities based on the services these facilities provide (primary, secondary and tertiary health services). We used the median fees of the different healthcare facility levels, for of the 3 types of wards (general ward, HCW and ICU) reported in the UPFS.

Table S3. Facility fees for each ward (2020 USD)

	**General ward**	**High care ward**	**Intensive care unit**
**Facility fee per patient per day**	64.57	196.55	516.36

References

1. Department of Health Province of Kwazulu Natal. ZNB 5769/2016-H: The supply and delivery of patient clothing and hospital linen for various institutions [Internet]. Department of Health Province of KwaZulu Natal; 2017 [cited 2020 Apr 8]. Available from: http://www.kznhealth.gov.za/Tenders/Awarded/2017/ZNB5769.2016-H.pdf
2. The Paramedic Shop. Pillow case [Internet]. 2020 [cited 2020 May 14]. Available from: https://www.paramedicshop.co.za/products/standard-pillow-case?_pos=2&_sid=e2541fc10&_ss=r
3. The Paramedic Shop. Standard Pillow [Internet]. 2020 [cited 2020 May 14]. Available from: https://www.paramedicshop.co.za/products/standard-pillow?_pos=4&_sid=e2541fc10&_ss=r
4. COVID-19 Cost Working Group. National COVID-19 Budget Model South Africa. 2020 Apr.
5. Afrimedics. CONTEC Infusion Pump SP800 [Internet]. Infusion Pump and Syringe Drivers. 2020 [cited 2020 May 25]. Available from: https://afrimedics.co.za/product/contec-infusion-pump-sp800/
6. Afrimedics. Contec CMS6500 Multi-parameter Vital Signs Monitor [Internet]. Patient Monitor. 2020 [cited 2020 May 25]. Available from: https://afrimedics.co.za/product/contec-cms6500-multi-parameter-vital-signs-monitor/
7. Afrimedics. Lovego G3 portable oxygen concentrator for 7LPM oxygen therapy [Internet]. Oxygen products and Accessories. 2020. Available from: https://afrimedics.co.za/product/lovego-g3-portable-oxygen-concentrator-for-7lpm-oxygen-therapy/
8. Afrimedics. Askir 20 Portable Surgical Suction Unit [Internet]. Surgical suction machine. 2020 [cited 2020 May 28]. Available from: https://afrimedics.co.za/product/askir-20-portable-surgical-suction-unit/
9. Afrimedics. 10L Oxygen Cylinder only [Internet]. Oxygen products and Accessories. 2020 [cited 2020 May 25]. Available from: https://afrimedics.co.za/product/10l-oxygen-cylinder-only/
10. Afrimedics. PVC Resuscitator – Adult [Internet]. Resuscitators & Resuscitator spares. 2020 [cited 2020 May 25]. Available from: https://afrimedics.co.za/product/pvc-resuscitator-adult/
11. World Health Organization. WHO COVID-19 Essential Supplies Forecasting Tool (ESFT) [Internet]. Available from: https://www.who.int/publications/m/item/covid-19-essential-supplies-forecasting-tool
12. MASHA, HE2RO, SACEMA. Estimating cases for COVID-19 in South Africa: Assessment of alternative scenarios. National Institute for Communiable Diseases; 2020 Sep.
13. Afrimedics. Laryngoscope Fibre Optic 4 Blade Set [Internet]. Laryngoscope. [cited 2020 Dec 9]. Available from: https://afrimedics.co.za/product/laryngoscope-fibre-optic-4-blade-set/
14. Guidelines for Intensive Care In South Africa. South Afr J Anaesth Analg. 2006 Jan;2006(1):7.1-7.8.
15. Balkhy HH, Perl TM, Arabi YM. Preventing healthcare-associated transmission of the Middle East Respiratory Syndrome (MERS): Our Achilles heel. J Infect Public Health. 2016 May 1;9(3):208–12.
16. Department of Public Service and Administration. Salary scales, with translation keys, for employees on salary levels 1 to 12 and those employees covered by Occupation Specific Dispensations (OSDs). 2020.
17. Driggin E, Madhavan MV, Bikdeli B, Chuich T, Laracy J, Biondi-Zoccai G, et al. Cardiovascular Considerations for Patients, Health Care Workers, and Health Systems During the COVID-19 Pandemic. J Am Coll Cardiol. 2020 May 12;75(18):2352–71.
