# Supplementary material for: Inpatient Care Costs of COVID-19 in South Africa’s Public Healthcare System
Source: Int J Health Policy Manag. 2021 Apr 25;11(8):1354–61. doi: 10.34172/ijhpm.2021.24 (PMC9808349; doi:10.34172/ijhpm.2021.24)
Supplement: Supplementary file 4 — contains Figures S1-S6. [file ijhpm-11-1354-s004.pdf]

Supplementary file 4

Scenario analysis results

Figures S1-S6 present the results of each of the series of scenario analyses, in the form of tornado diagrams.

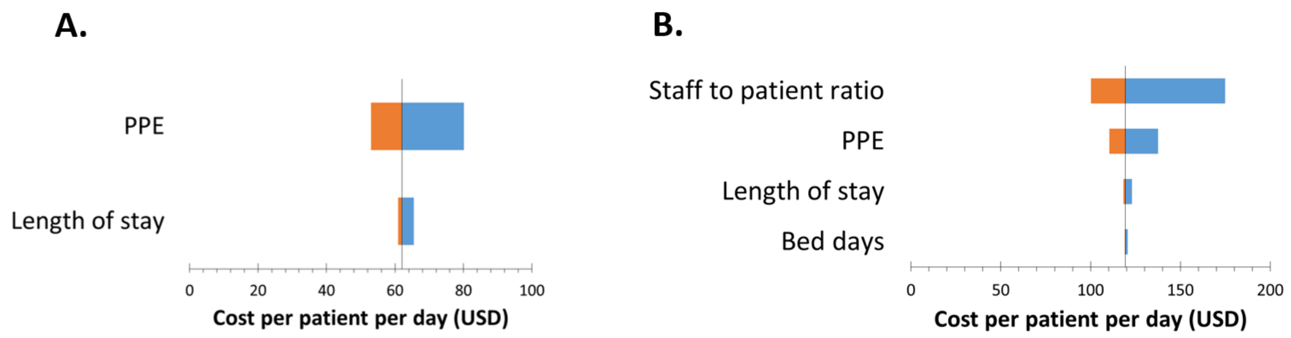

Figure S1 Scenario analysis for general wards with no oxygen - financial cost (A) and economic cost (B) per patient per day (excluding facility fee)

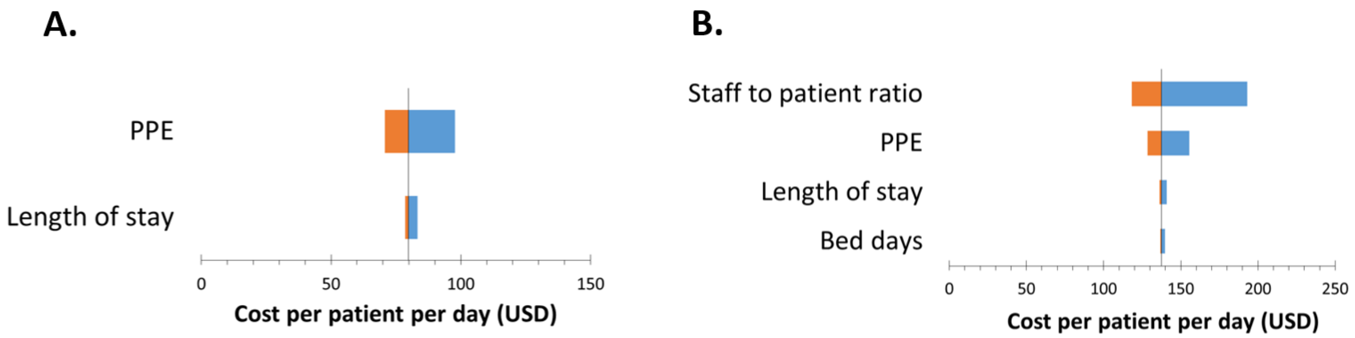

Figure S2. Scenario analysis for patients in general wards with oxygen - financial cost (A) and economic cost (B) per patient per day (excluding facility fee)

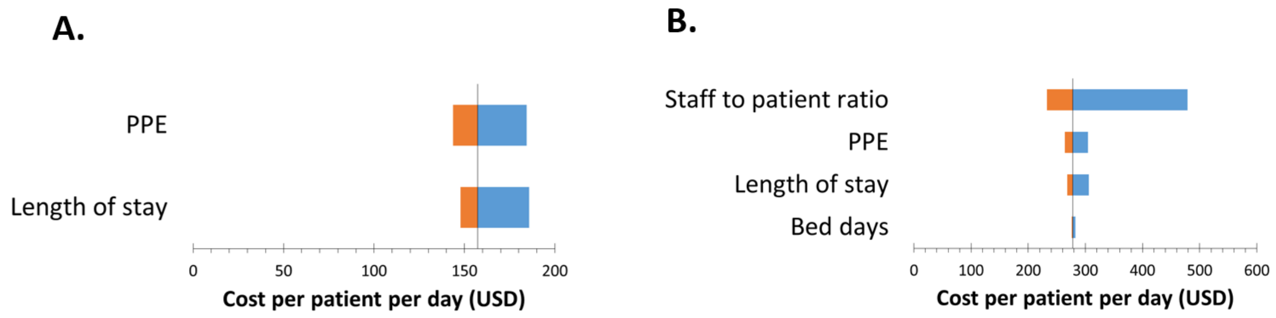

Figure S3. Scenario analysis for high care wards with high flow nasal oxygen - financial cost (A) and economic cost (B) per patient per day (excluding facility fee)

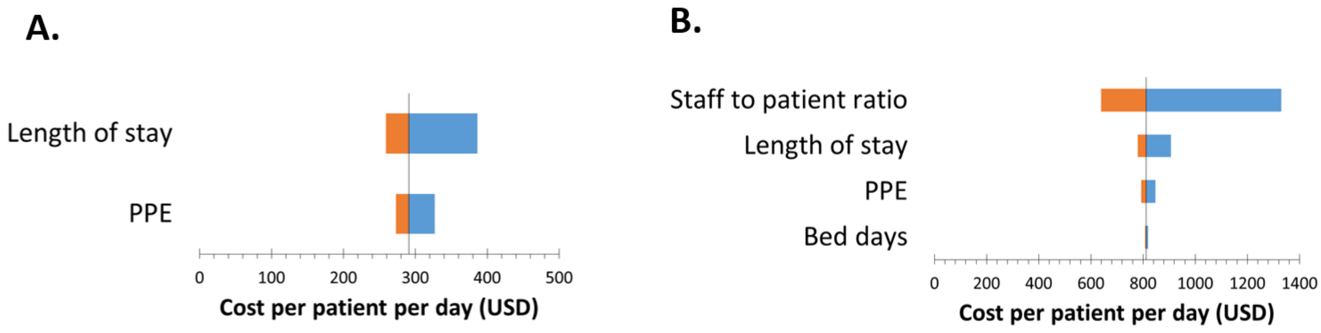

Figure S4. Scenario analysis for ICU with CPAP - financial cost (A) and economic cost (B) per patient per day (excluding facility fee)

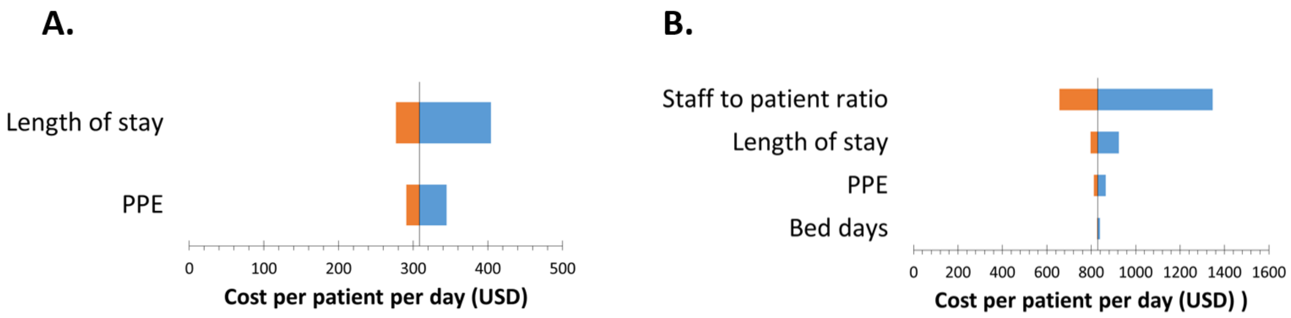

Figure S5. Scenario analysis for ICU with NIV - financial cost (A) and economic cost (B) per patient per day (excluding facility fee)

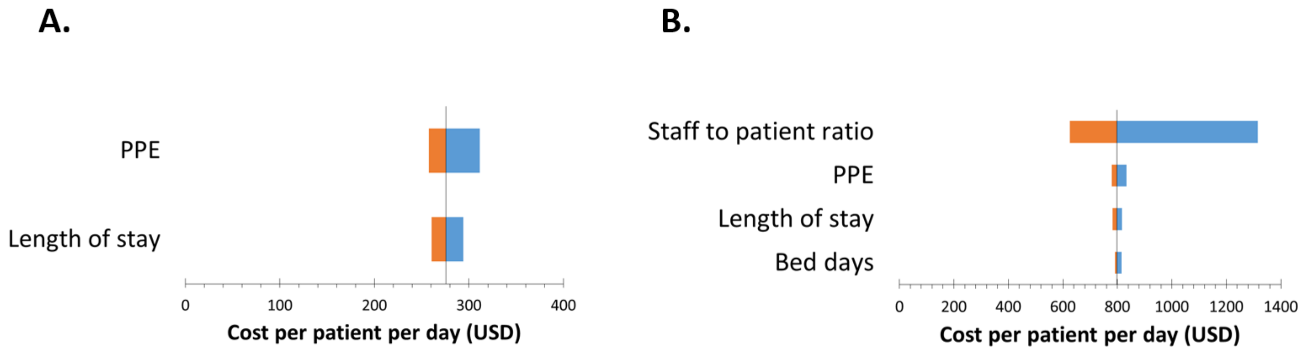

Figure S6. Scenario analysis for ICU with invasive mechanical ventilation - financial cost (A) and economic cost (B) per patient per day (excluding facility fee)
